# Conformational Dynamics of Phytoglobin BvPgb1.2 from *Beta vulgaris* ssp. *vulgaris*

**DOI:** 10.3390/ijms24043973

**Published:** 2023-02-16

**Authors:** Simon Christensen, Olof Stenström, Mikael Akke, Leif Bülow

**Affiliations:** 1 Division of Pure and Applied Biochemistry, Center for Applied Life Science, Department of Chemistry, Lund University, 22100 Lund, Sweden; 2 Division of Biophysical Chemistry, Center for Molecular Protein Science, Department of Chemistry, Lund University, 22100 Lund, Sweden

**Keywords:** sugar beet, hemoglobin, phytoglobin, subunit interactions, NMR relaxation, amino acid labeling

## Abstract

Plant hemoglobins, often referred to as phytoglobins, play important roles in abiotic stress tolerance. Several essential small physiological metabolites can be bound to these heme proteins. In addition, phytoglobins can catalyze a range of different oxidative reactions in vivo. These proteins are often oligomeric, but the degree and relevance of subunit interactions are largely unknown. In this study, we delineate which residues are involved in dimer formation of a sugar beet phytoglobin type 1.2 (BvPgb1.2) using NMR relaxation experiments. *E. coli* cells harboring a phytoglobin expression vector were cultivated in isotope-labeled (^2^H, ^13^C and ^15^N) M9 medium. The triple-labeled protein was purified to homogeneity using two chromatographic steps. Two forms of BvPgb1.2 were examined, the oxy-form and the more stable cyanide-form. Using three-dimensional triple-resonance NMR experiments, sequence-specific assignments for CN-bound BvPgb1.2 were achieved for 137 backbone amide cross-peaks in the ^1^H-^15^N TROSY spectrum, which amounts to 83% of the total number of 165 expected cross-peaks. A large proportion of the non-assigned residues are located in α-helixes G and H, which are proposed to be involved in protein dimerization. Such knowledge around dimer formation will be instrumental for developing a better understanding of phytoglobins’ roles in planta.

## 1. Introduction

Hemoglobins (Hb) are essential for almost all forms of life. These heme-equipped proteins are not limited to mammals, but are found in a wide range of living organisms, spanning from vertebrates and plants to protozoans and bacteria [1]. Several metabolic roles have been attributed to Hbs in addition to binding a variety of compounds including oxygen, carbon monoxide (CO), nitric oxide (NO), hydrogen sulfide (H_2_S), hydroxylamine (NH_2_OH), and several other small organic molecules [2,3]. Hbs are also able to catalyze a range of oxidative reactions thereby suggesting that these proteins show a large diversity of physiological functions [4,5,6].

Particularly plant Hbs have attracted substantial scientific attention during the last decade. However, already in the 1930s, the first plant Hb was discovered, a symbiotic Hb from soybean [7]. Since then, different classes of plant Hbs, also called phytoglobins (Pgb) [8], have been identified. Several physiological functions have been proposed for these proteins, including a roles in adaptation and survival during hypoxia [9,10,11], metabolism of nitrogen [12,13], and hormone/signaling pathways [13,14,15].

Hbs are composed of a heme b prosthetic group inserted within an α-helical protein structure composed of eight helices, A–H. The binding between the heme group and the globin chains differ. Class I Pgb proteins have a low hexa-coordination equilibrium constant (K_H_), which describes the binding constant of the distal histidine and hence the equilibrium of the penta- and hexa-coordinated states [16]. The low K_H_ results in high affinity for oxygen, which is optimally designed for oxygen-dependent nitric oxide scavenging in low oxygen-containing environments [17,18]. Furthermore, the slow oxygen dissociation rate makes class I Pgs unlikely to function as oxygen transporters. Instead, Class I Pgb play an important role in electron transport due to their high oxygen affinity and redox potential, which serve to maintain the energy status of the cell during oxygen deprivation [7,18]. However, this reasoning is not universally accepted. Instead, recent research of Pgbs from sugar beet suggests that even if the Hbs have intrinsic properties to catalyze certain oxidative reactions, the location and concentration of ligands, such as oxygen, NO, and nitrite will ultimately determine their activity [13]. 

Over the years, the biennial sugar beet (*Beta vulgaris* spp. *vulgaris*) has gathered substantial interest due to its socioeconomical importance for both sugar and energy production. In this caryophyllidae, three different Pgbs have been found (BvPgb1.1, BvPgb1.2, and BvPgb2). Their gene expression pattern differs depending on, e.g., the growth phase and photoperiods of the plant [3]. In a study by Leiva-Eriksson et al. [13], the characteristics of these relatively recently discovered Pgbs were determined and compared to existing data for Pgbs from both eudicots and monocots. Here, it was found that BvPgb1.2 (Pgb class 1) has a lower equilibrium binding constant for oxygen (K_O2_) compared to the common model Hb protein in *Arabidopsis*, AtHb1, and to monocotic Pgbs from barley, rice, and maize [13]. Furthermore, BvPgb1.2 also exhibited a lower hexa-coordination constant (K_H_) in comparison with proteins found in *Arabidopsis thaliana* and the mentioned monocots.

In addition, the class II Pgb from sugar beet (BvPgb2) was recently heterologous expressed in yeast, *A. thaliana* and tomato (*Solanum lycopersicum*). In the case of *A. thaliana*, such overexpression conferred improved drought and osmotic stress tolerance. The enhanced levels of BvPgb2 in tomato both increased iron levels in leaves and made the plants more resistant to drought-induced withering [19]. Since tomato is the most consumed horticultural crop worldwide, there is a strong driving force to lower its high water and irrigation requirements and make the crop more resilient to external abiotic stress factors [19]. In addition, when this protein was overexpressed in field cress (*Lepidium campestre)*, an increased oil content was observed [20]. These results on BvPgbs have provided important information on the diversity of Pgbs. The metabolic roles of these proteins and other Hbs in crops have indicated their potential to improve agricultural performances [21,22]. Furthermore, food quality in terms of enhanced iron levels may promote the use of these proteins as additives in various products.

Due to growing importance of Pgbs it is essential to further delineate possible structure-function relationships to explain their physiological functions. We recently solved the three-dimensional structure of BvPg1.2 [23] in its monomeric form. Since this Pgb is largely present in a dimeric form, it is essential to gain more knowledge on the subunit interactions present in the protein. Nuclear magnetic resonance (NMR) spectroscopy is a powerful tool to examine molecular structure, dynamics and interactions [24]. In the field of protein science, NMR spectroscopy hence provides additional information on dynamics that complements structural information obtained by X-ray crystallography. NMR relaxation methods probe internal motions within the molecule that are often essential for function [25,26]. One common obstacle in protein NMR is the size limitation caused by spectral overlap among signals and line-broadening, both of which increase with molecular size. However, by labeling the protein with stable isotopes (^15^N and ^13^C) the overlap problem can be mitigated, and labeling with ^2^H significantly reduces line-broadening, enabling NMR studies of proteins up to 40–50 kDa [27,28].

In this study, we report on the conformational dynamics of BvPgb1.2 in its cyanide-bound form, based on backbone ^15^N longitudinal (R1) and transverse (R2) NMR relaxation rate measurements. In addition, we compare the spectral characteristics of the oxy- and cyanide-bound forms. The main objective was to expand the current knowledge regarding dimer–dimer interactions and identifying dynamic motions/flexible regions in this class of Pgbs. The results clearly indicate which protein regions are involved in dimer formation and highlighting dynamic movements in BvPgb1.2. Since BvPgbs can interact with a range of other biomolecules in vivo, including nucleic acids, lipids, and other proteins, the main objective was to further dissect the subunit interaction areas which most likely are involved in such interactions.

## 2. Results and Discussion

**Figure 1:**
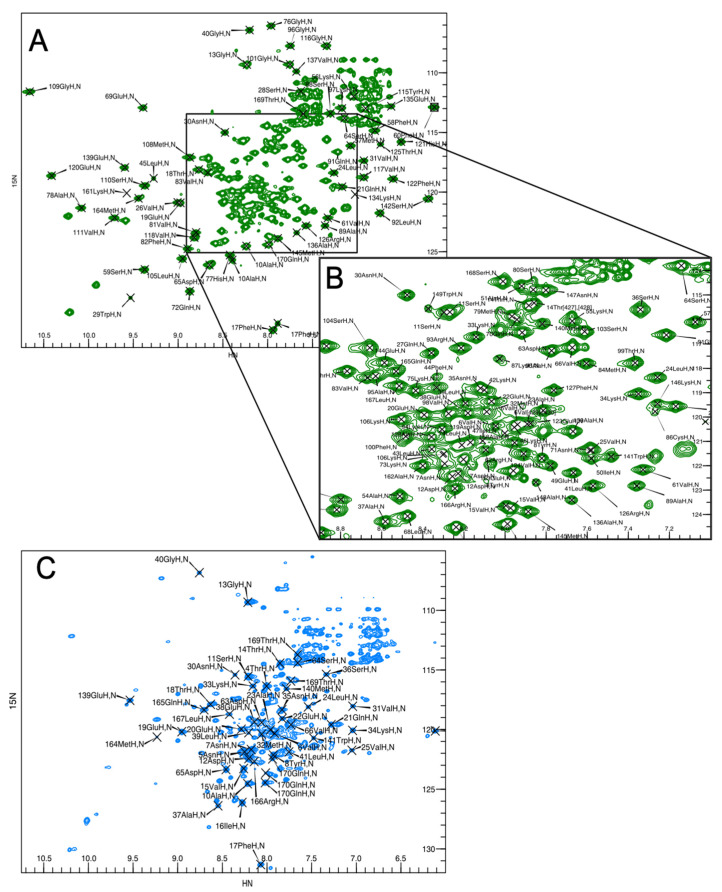
^1^H–^15^N-TROSY spectra with assignments for BvPgb1.2 in complex with cyanide (**A**,**B**) and oxygen (**C**). The spectra were acquired at 298 K and a magnetic field strength of 18.8 T. Panel B is a close-up of the boxed area in panel A.

We assigned the NMR spectrum for CN-bound BvPgb1.2 using three-dimensional triple-resonance experiments (see Section 3). We achieved sequence-specific assignments for the cross-peaks from 137 backbone amides in the ^1^H-^15^N TROSY spectrum, Figure 1, which amounts to 83% of the total number of 165 expected cross-peaks (171 residues minus 6 prolines). For an additional 13 residues, we could assign one or several of the ^13^C_α_, ^13^C_β,_ and ^13^C’ (carbonyl) chemical shifts, while 15 non-proline residues remain unassigned. There are several unassigned peaks in the ^1^H-^15^N TROSY spectrum indicating that there are one or a few broadened peaks separating the unassigned from assigned peaks.

**Figure 2:**
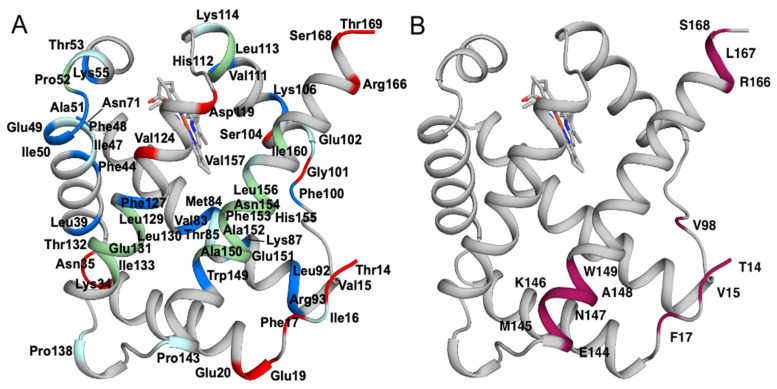
(**A**) High R_2_ (blue), high R_1_ (red), missing (green), and partial (cyan) assignments and (**B**) residues showing two conformations or line broadening (purple) color coded on the structure of BvPgb1.2.

By comparison, in the ^1^H-^15^N TROSY spectrum of oxygen-bound BvPgb1.2, we could assign only 48 out of the 165 expected cross-peaks from backbone amide groups. The lack of assignment can be explained by the paramagnetic property of the ferrous (Fe^II^) heme, which is in a high-spin state when bound to oxygen [29,30], causing significantly increased relaxation of the nuclear spins. In contrast, CN-bound BvPgb1.2 is in the diamagnetic, low-spin ferric state (Fe^III^) [31], resulting in slower relaxation and improved NMR spectra. This effect is further illustrated by Figure 2, which shows assigned residues color coded in blue on the structure; the assigned residues are those located furthest away from the heme-group where oxygen is bound [32]. 

In what follows, we focus our report on the dimerization of CN-bound BvPgb1.2 and its conformational dynamics. 

### 2.1. NMR Spectroscopy Provides Evidence for Dimerization of CN-BvPgb1.2

All residues with missing assignment or partial assignment in CN-bound BvPgb1.2 are listed in Table 1 and mapped onto the structure in Figure 2A. One notable missing assignment is the proximal histidine H112, which coordinates the iron of the heme group together with the distal histidine H77. Assignments are also missing for residues 129–133 located in α-helix G, and for residues 150–156 in the adjacent helix H. The preceding residues 144–149 appear significantly broadened or have a shoulder, suggesting that they are present in two conformations. Moreover, residues in the N-terminal segment, located next to helix H, also show evidence of populating multiple conformations: V6 shows three peaks in the ^1^H-^15^N TROSY spectrum and residues 7–17 all show two peaks, except for G13, which was assigned to a single peak, and I16, which is missing in the spectrum. Residues showing multiple sets of peaks or peak broadening are listed in Table 1 and mapped onto the structure in Figure 2B. Notably, residues with conformational heterogeneity map to a contiguous region of the protein structure, which has previously been implicated in mediating dimerization of plant Hbs. The dimer dissociation constant (K_d_) has been determined to 86 μM for rice Hb1 in the ferric state [33] (63.4% sequence identity compared to BvPgb1.2). Based on this value, we estimate that the fraction of monomeric protein in the NMR sample of CN-bound BvPgb1.2 is around 20% of the total protein concentration. Furthermore, the dimer dissociation constants for phytoglobins from *Parasponia andersonii*, *Trema tomentosa, and Arabidopsis thaliana* (72.2, 74.1, and 74.4% sequence identity, respectively) have been determined and shown to be mutually similar with K_d_ ~1 μM [34,35,36]. Using this K_d_ value, one would predict that there is only ~3% monomer in the BvPgb1.2 NMR sample. 

A large proportion of the non-assigned residues is situated in α-helixes G and H, which in other Hbs are involved in dimerization [33]. A likely explanation for the missing assignments is exchange broadening due to transient dimerization or conformational exchange at the dimer interface. Chemical exchange has been observed previously in the corresponding structural region of other Hbs, and has been explained by a sliding motion involving the two protomers or by slow diffusion of low-molecular weight molecules, such as O_2_, NO or CO, in ‘tunnels’ forming between helices G and H [37,38]. The latter explanation is unlikely in the present case, given the sample conditions, while the former is a possible explanation for the conformational heterogeneity observed in the NMR spectra. Note that exchange due to transient dimerization or sliding motions are not mutually exclusive. Several NMR studies have also observed exchange in the F-helix [37,39]. It is certainly plausible that the four unassigned residues in helix F of CN-bound BvPgb1.2 might be broadened by conformational exchange. The exchange timescale(s) are not well defined: the observation of multiple peaks (or shoulders) suggests that the underlying exchange is slower than the difference in chemical shift between the alternative states, whereas the exchange broadening beyond detection might suggest a relatively fast process, or that the chemical shift difference in these cases is smaller. As mentioned above, it is possible that there are two distinct processes active: transient dimerization, which might be fast, and local conformational exchange, which might be slower, or vice versa. We address this issue next using ^15^N spin relaxation methods. 

### 2.2. ^15^N Relaxation Confirms Dimerization and Identifies Flexible Regions

**Figure 3:**
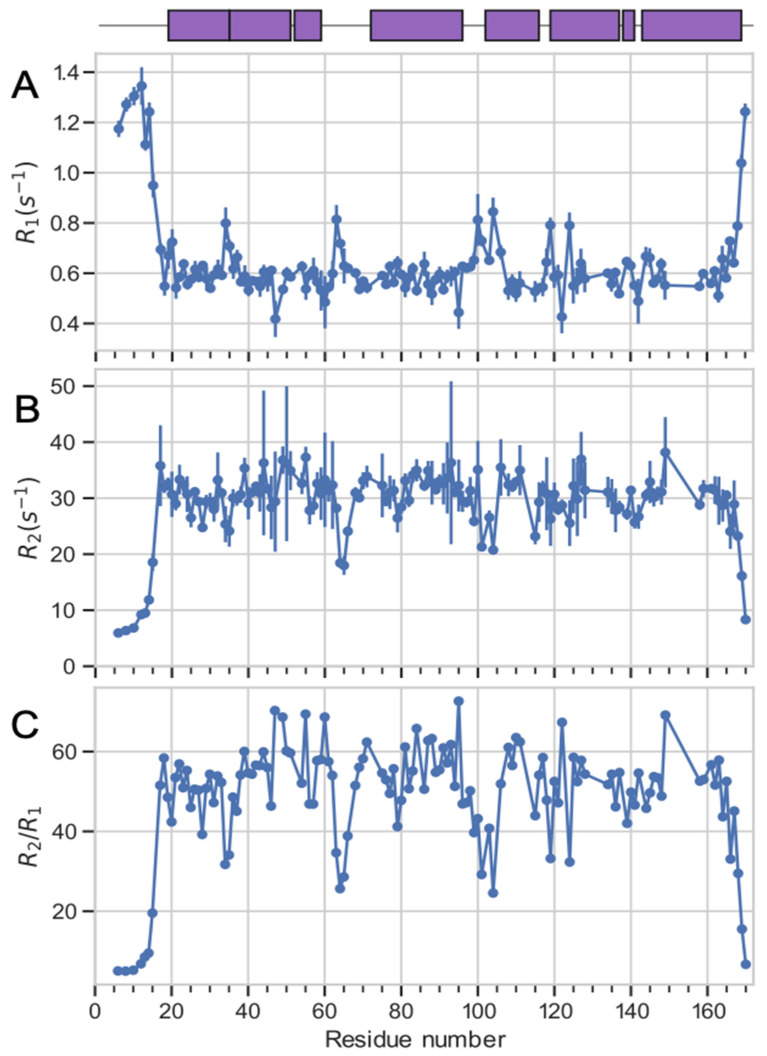
NMR relaxation data R_1_ (**A**), R_2_ (**B**), and R_2_/R_1_ (**C**) measured at 600 MHz, plotted against residue number. Secondary structure elements along the sequence are shown on top, where alpha helices are represented by purple boxes and loop regions by black lines.

To characterize the conformational dynamics of CN-bound BvPgb1.2, we measured longitudinal (R_1_) and transverse (R_2_) ^15^N relaxation rate constants that probe motions on the picosecond to nanosecond and millisecond time scales. Out of the 137 assigned residues, 7 residues (N7, S11, Q72, K73, L74, L105, and E123) were excluded from further analysis due to severe spectral overlap. The remaining 130 residues yielded high quality relaxation data (Figure 3A,B), albeit with relatively large error bars for a handful of residues with higher than average values of R_2_ (Figure 3B), as might be expected due to the increased line-broadening. 

We determined the rotational correlation time (τ_c_) from the residue-specific ratios of R_2_/R_1_ and the structure of BvPgb1.2 (pdbID: 7ZOS [23]), using the program ROTDIF (Table 2), based on a dataset trimmed by removing all R_2_/R_1_ values outside of one standard deviation from the mean [40]. The trimmed data set comprises 104 residues. The best fitting diffusion model is anisotropic with τ_c_ = 22.8 ns, an anisotropy of 0.84, and a rhombicity of 0.63. We compared τ_c_ determined by ROTDIF with τ_c_ calculated using HYDRONMR based on the monomeric structure of BvPgb1.2 (PDB-id: 7ZOS [23]) and the dimeric structure of BvPgb C86A (PDB-id: 7Z1U [23]). HYDRONMR predicts τ_c_ values of 11.3 ns and 28.9 ns for the monomeric and dimeric structures, respectively, in agreement with correlation times observed for other Hbs [39,41]. The anisotropy and rhombicity was 0.83 and 0.40 for the monomeric structure and 1.44 and 0.12 for the dimeric. Together, these results suggest that CN-BvPgb1.2 exists primarily in dimeric form in solution at the present concentration, as is also found for other phytoglobins [29,31,42]. This conclusion is also in agreement with the weak intensity of the minor cross-peaks in the NMR spectrum, which indicate that the monomer population must be low, if the system is in slow exchange. Based on results from this and previous work [23], we suggest hydrogen bonding primarily between T53-E120 in opposing dimers but H121-E120 could also interact in this way. Moreover, a hydrophobic cluster between I47, I50, V124 and F127 aids in dimer formation through hydrophobic interactions, even though additional residues with similar properties might facilitate quaternary stabilization as well. All these residues are primarily located in the B/C loop and G helix, in line with the previous hypothesis [36]. 

The relaxation data show increased flexibility for some 25 residues with low values of R_2_/R_1_ (Figure 3C). The N and C termini are both highly flexible. Other positions with low R_2_/R_1_ ratios include residues 28, 34–35, 63–66, 99, 101, 104, 119, and 124. For most of these residues, the increased flexibility can be explained by structural features: K34 and N35 are located in the kink region between α-helices A and B; 63–66 are situated between helixes C and E in a region that is lacking electron density in the crystal structure, indicating high flexibility; T99, F100, and G101 reside in the loop region between helixes E and F; S103 and S104 are situated at the N-terminal end of α-helix F. Two residues, S28 and V124, with increased flexibility are more difficult to explain in terms of structure, as they are located in the middle of α-helices A and G, respectively. 

Elevated R_2_/R_1_ ratios are observed for residues L39, I47, E49, I50, K55, F60, V81, M84, K87, S88, Q91, R93, M108, S110, V111, F122, and W149. Residues L39, I47, E49, and I50 are all located in non-central parts of helix B, while K55 is in helix C, close to the heme group, but not directly interacting with it. F60 is located at the beginning of the loop directly following helix C. V81, M84, K87, S88, Q91, and R93 are all located on the same side of helix E, consecutively separated by one turn of helix and connected by hydrogen bonds. V81 is located one turn of helix away from the heme-coordinating H77. It is possible that the pattern of R_2_/R_1_ observed for these residues suggests transient bending of the helix. A similar pattern is observed for residues M108, S110, and V111 in helix F, which also harbors H112, one of the other heme-coordinating residues. Furthermore, residues V81, M84, M108, V111, and F122 have their side chains lining the heme-binding pocket. Taken together, these results might suggest that the heme-binding pocket is undergoing some type of breathing motions, accompanied by transient bending or fraying of the neighboring helices. 

In a recent study, we investigated the structural and functional roles of C86 in BvPgb1.2 by comparing the wild-type protein with a C86A mutant [23]. Wild-type BvPgb1.2 crystallized as a monomer, while the C86A mutant (PDB-id:7Z1U [23]) crystallized as a dimer, with only minor structural differences observed between the two variants. The dimeric structure emphasizes the importance electrostatic and hydrophobic interactions between the B/C and G helices in the neighboring subunit [36]. Our results showed that while these two variants have very similar three-dimensional structures, the autoxidation rate is increased for the C86A mutant, which also has a lower peroxidase activity. In addition, Y115 has been suggested to control the redox stability of the iron together with C86 [43,44]. While the aromatic ring of Y115 is directly interacting with the heme, the side chain of C86 is located between helices A and E and is pointing away from the heme towards the surface of the protein. Both these residues have relaxation rates similar to the average value observed for other central residues in helices, indicating that their dynamics do not stand out. However, both residues might still be affected by the dynamics observed in helices E and F, described above: it is likely that bending or fraying of helix E would affect the solvent accessibility of the C86 sulfhydryl group, thereby influencing its oxidation state. Similarly, conformational dynamics of helix F might well modulate the interactions between the Y115 side chain and the heme. 

## 3. Materials and Methods

### 3.1. Protein Expression and Purification

#### 3.1.1. Construction and Transformation of Expression Constructs

The gene for *Beta vulgaris* spp. *vulgaris* Pgb with GenBank Accession KF549981 (BvPgb1.2) [13] was custom synthesized by Epoch Biolabs (Missouri City, TX, USA). Recombination based cloning and transformation were carried out according to manufacturer’s instructions (Gateway^TM^, Invitrogen, Waltham, MA, USA). The gene contained attB1 and attB2 sites at the 5′ and 3′ ends, respectively. The genes were individually cloned into a pET-DEST42 plasmid. The final expression vector was transformed into BL21-DE3 *E. coli* cells.

#### 3.1.2. Expression of Triple-Labeled Protein in Minimal Media

The cells containing the expression vectors were grown in conventional M9 minimal media containing ^15^N- or ^13^C/^15^N-labeled components. The adaptation of *E.coli* BL21-DE3 cells were based on previous work with some modifications [45]. (i) Cells were inoculated in LB medium overnight. (ii) 15 mL non-deuterated M9 medium was inoculated with cells from the overnight culture. The cells were grown ~8 h and centrifuged 5 min at 10,000 rpm, followed by resuspension in fresh M9 media for another 8 h. (iii) Cells were centrifuged as before and suspended in M9 medium containing 70% D_2_O and grown for ~8 h or until sufficient cell mass was obtained. This step was done twice. (iv) Cells were suspended in M9 medium containing 99.8% D_2_O in the same way as before. This was done twice. (v) Cells were suspended in M9 medium containing 0.8% *w*/*v* ^13^C-glucose, 2 g/L ^15^NH_4_Cl, and 99.8% D_2_O. This was done three times, where a 15% glycerol stock of adapted cells were frozen and kept. The final cultivation volume was 3 L of triple-labeled M9 medium and shake flasks were inoculated using adapted cells from step v. 

Cells containing the expression vectors were grown in triple-labeled minimal medium containing 100 µg/mL carbenicillin at 37 °C and 150 rpm until A600 ≥ 2. Expression of BvPgb1.2 was then induced by adding 0.5 mM IPTG and 0.3 mM δ-aminolevulinic acid. The cells were also briefly bubbled with CO to obtain a stable CO-BvPgb when expressed. After induction, cells were grown for 48 h at 22 °C, 150 rpm, then collected by centrifugation, and finally frozen at −80 °C until used.

#### 3.1.3. Cell Harvesting and Protein Extraction

Cell harvesting and protein extraction for the triple-labeled protein was conducted using previously described methods [13].

#### 3.1.4. Protein Purification

The extracted supernatant was dialyzed twice in 4-L 50 mM Tris-HCl pH 8.5 buffer during 12h before protein purification. Dialyzed lysates were then passed through 1.2 µm filter (Sarstedt, Nümbrecht, Germany). BvPgb1.2 were lastly separated and purified from the lysates through a two-step chromatography process consisting of an initial anion-exchange step followed by a hydrophobic interaction chromatography step. Both steps were performed on an ÄKTA™ Avant (Cytvia Life Science, Singapore) chromatography system. All buffers used for chromatography were pH adjusted and degassed, and all solutions were passed through a 0.45 µm filter before being loaded onto columns. Filtered lysates were first loaded onto a Q-Sepharose FF column (Cytvia Life Science) and eluted with three column volumes (CV) of 50 mM Tris-HCl pH 8.5 and 200 mM NaCl. The eluted BvPgb1.2 fractions were pooled and concentrated using 10 kDa Vivaspin^®^ 20 mL ultrafiltration units (Vivascience, Stonehouse, UK) before adding ammonium sulfate (NH_4_)_2_SO_4_ to a final concentration of 1 M. The fractions were then loaded onto a Butyl-Sepharose HP column (Cytvia Life Science) pre-equilibrated with 50 mM Tris-HCl pH 8.5 buffer and 1.5 M (NH_4_)_2_SO_4_. 

BvPgb1.2 were then eluted with a three CV linear gradient ranging from 1 M to 0 M (NH_4_)_2_SO_4_ in 50 mM Tris-HCl pH 8.5 buffer. The final BvPgb1.2 fractions had an absorbance ratio at 412 nm and 280 nm (A412/A280) in the range 2.5–3, corresponding to >98% purity. BvPgb1.2 fractions were lastly concentrated using 10 kDa Vivaspin^®^ 20 mL ultrafiltration units (Vivascience) and stored at −80 °C. When examined by analytical size exclusion chromatography the protein samples were identified as dimers [23].

### 3.2. Protein Sample Preparation

To prepare the cyanide form of BvPgb1.2, the purified protein was dialyzed in 10 mM potassium ferricyanide and 1 mM potassium cyanide dissolved in 50 mM MOPS for 8 h in 0.5 L solution; this process was performed twice. The cyanide-protein solution was passed through a PD10 column (Cytvia Life Science) to remove excess cyanide and the protein was concentrated using 10 kDa Vivaspin^®^ 20 mL ultrafiltration units (Vivascience). The oxy form of BvPgb1.2 was generated by passing the purified Hb through a PD10 column (Cytvia Life Science) in order to equilibrate with the oxygenated buffer (50 mM MOPS). 

### 3.3. NMR Spectroscopy and Data Analysis

#### 3.3.1. NMR Sample Preparation

NMR samples of CN-BvPgb1.2 contained 1 mM protein in 400 μL 50 mM MOPS pH 7.4 with 8% D2O added for the field-frequency lock system on the NMR spectrometer. 

#### 3.3.2. Triple-Resonance NMR Experiments and Data Analysis for Backbone Assignment

Three-dimensional triple-resonance ^1^H/^13^C/^15^N NMR experiments for backbone resonance assignments were carried out at 298 K on a Bruker Avance HDIII 800 MHz spectrometer, equipped with a TCI 800S7 H-C/N-D-03 Z probe. A series of ^1^H–^15^N TROSY-based three-dimensional 1H-detected experiments with ^2^H decoupling were acquired using targeted acquisition [46] and random non-uniform sampling. The experiments included HNCO (25% and 30% sampling density), HN(CO)CA (12% and 13.9% sampling for the CN-bound and oxygen-bound samples, respectively), HNCA (12% and 12.9%), HNCACB (23% and 26%), HN(CO)CACB (24% and 26%), and HN(CA)CO (11% and 13.1%). The data were processed using the compressed sensing IRLS algorithm in MddNMR [47,48]. Residue-specific assignment was carried out using the CcpNmr Analysis software suite [49].

#### 3.3.3. ^15^N NMR Relaxation Experiments and Data Analysis

^1^H–^15^N TROSY-based ^15^N R_1_ and R_2_ relaxation experiments targeting the backbone amides [50] were performed at a static magnetic field strength of 14.1 T and a temperature of 298 K. Spectral widths were 14.4 ppm and 30 ppm, covered by 2048 and 256 points in the ^1^H and ^15^N dimensions, respectively. Relaxation decays were recorded using 10 delays with times ranging from 0.020–1.2 s and 0–220 ms for R1 and R2, respectively. Spectra were processed using NMRPipe [51] and a processing protocol including zero filling to twice the number of data points in both dimensions, solvent filter, and a squared cosine apodization. Relaxation rates were calculated using PINT, which can resolve overlapped peaks using line fitting [52]. Peak intensities were evaluated using a weighted sum of Gaussian and Lorentzian lineshape functions. Error estimation of the fitted relaxation rates was performed using the jack-knife procedure.

HydroNMR [53] calculations were performed using version 7c through NMRBox [54] at 14.1 T and with atomic element radius (AER) of 3.1 Å. We determined the τ_c_ of the BvPgb1.2 by fitting the rotational diffusion tensor to the ^15^N R_1_ and R_2_ values using the JAVA version of Rotdif 3.1 [55,56]. The ^1^H–^15^N bond vector orientations were taken from the structure of BvPgb1.2 to which hydrogens were added using the AddH function in Chimera [57].

## 4. Conclusions

In this study, we investigated the conformational dynamics in sugar beet hemoglobin BvPgb1.2, the first phytoglobin to be characterized using NMR in an extensive way. A proportion of the non-assigned residues is situated in α-helixes G and H, where tunnel forming between these helices might occur, leading to diffusion of low-molecular weight molecules. Correlation time determination using ROTDIF and HYDRONMR revealed similar correlation times compared to a dimeric phytoglobin, suggesting that BvPgb1.2 exists mainly as a dimer for these conditions. Results from ^15^N relaxation indicated a dynamic motion of the heme pocket which might occur in bending and fraying of the neighboring helices. However, previously studied residues that might be important for redox environment and electron acceptors around the heme pocket (C86 and Y115) displayed relaxation rates that did not stand out from average values, although it is still possible that the bending of the E and F helices might affect solvent accessibility and modulate interactions for these residues. Taken together, the results revealed important clues into the dynamic motions of these proteins and how these conformations might lead to subunit interactions in phytoglobins.

## Figures and Tables

**Table 1:** List of residues with partial assignments and missing assignments.

Partial assignments	N5 P9 I16 I47 F48 T53 P67 T85 E102 K114 P138 P143 V157
Missing assignments	M1 S2 F3 T4 P52 H112 L113 L129 L130 E131 T132 I133 A150 E151 A152 F153 N154 H155 L156 I160 P171
Resides with more than one conformation	V6 N7 Y8 A10 S11 D12 G13 T14 V15 F17 V98 E144 M145 K146 N147 A148 W149 R166 L167 S168

**Table 2:** Properties of diffusion tensor determined by ROTDIF and HYDRONMR.

	τc (ns)	Anisotropy	Rhombicity
ROTDIF	22.8	0.84	0.63
Monomer (pdbID: 7ZOS)	11.3	0.83	0.40
Dimer (pdbID: 7Z1U)	28.9	1.44	0.12

## Data Availability

Experimental data can be obtained from the corresponding auhors on reasonable request.

## References

[1] Jensen F.B., Fago A., Weber R., Perry S., Tufts B. (1998). Hemoglobin Structure and Function. Fish Phys.

[2] Vinogradov S.N., Moens L. (2008). Diversity of globin function: Enzymatic, transport, storage, and sensing. J. Biol. Chem..

[3] Leiva-Eriksson N., Pin P.A., Kraft T., Dohm J.C., Minoche A.E., Himmelbauer H., Bülow L. (2014). Differential expression patterns of non-symbiotic hemoglobins in sugar beet (*Beta vulgaris* ssp. *vulgaris*). Plant Cell Physiol..

[4] Mot A.C., Puscas C., Miclea P., Naumova-Letia G., Dorneanu S., Podar D., Dissmeyer N., Silaghi-Dumitrescu R. (2018). Redox control and autoxidation of class 1, 2 and 3 phytoglobins from Arabidopsis thaliana. Sci. Rep..

[5] Matilla A.J., Rodríguez-Gacio M.D.C. (2013). Non-symbiotic hemoglobins in the life of seeds. Phytochem.

[6] Athwal N.S., Alagurajan J., Andreotti A.H., Hargrove M.S. (2016). Role of Reversible Histidine Coordination in Hydroxylamine Reduction by Plant Hemoglobins (Phytoglobins). Biochemistry.

[7] Garrocho-Villegas V., Gopalasubramaniam S., Arredondo-Peter R. (2007). Plant hemoglobins: What we know six decades after their discovery. Gene.

[8] Hill R., Hargrove M., Arredondo-Peter R. (2016). Phytoglobin: A novel nomenclature for plant globins accepted by the globin community at the 2014 XVIII conference on Oxygen-Binding and Sensing Proteins. F1000Research.

[9] Taylor E.R., Nie X.Z., MacGregor A.W., Hill R.D. (1994). A cereal haemoglobin gene is expressed in seed and root tissues under anaerobic conditions. Plant Mol. Biol..

[10] Dordas C., Hasinoff B.B., Igamberdiev A.U., Manac’H N., Rivoal J., Hill R.D. (2003). Expression of a stress-induced hemolobin affects NO levels produced by alfalfa root cultures under hypoxic stress. Plant J..

[11] Athwal N.S., Alagurajan J., Sturms R., Fulton D.B., Andreotti A.H., Hargrove M.S. (2015). Electron self-exchange in hemoglobins revealed by deutero-hemin substitution. J. Inorg. Biochem..

[12] Ohwaki Y., Kawagishi-Kobayashi M., Wakasa K., Fujihara S., Yoneyama T. (2005). Induction of Class-1 Non-symbiotic Hemoglobin Genes by Nitrate, Nitrite and Nitric Oxide in Cultured Rice Cells. Plant Cell Physiol..

[13] Eriksson N.L., Reeder B.J., Wilson M.T., Bülow L. (2019). Sugar beet hemoglobins: Reactions with nitric oxide and nitrite reveal differential roles for nitrogen metabolism. Biochem. J..

[14] Hill R.D. (2012). Non-symbiotic haemoglobins—What’s happening beyond nitric oxide scavenging?. AoB Plants.

[15] Huang S., Hill R.D., Wally O.S., Dionisio G., Ayele B.T., Jami S.K., Stasolla C. (2014). Hemoglobin Control of Cell Survival/Death Decision Regulates in Vitro Plant Embryogenesis. Plant Physiol..

[16] Gupta K.J., Hebelstrup K.H., Mur L.A., Igamberdiev A.U. (2011). Plant hemoglobins: Important players at the crossroads between oxygen and nitric oxide. FEBS Lett..

[17] Trevaskis B., Watts R.A., Andersson C.R., Llewellyn D.J., Hargrove M.S., Olson J.S., Dennis E.S., Peacock W.J. (1997). Two hemoglobin genes in Arabidopsis thaliana: The evolutionary origins of leghemoglobins. Proc. Natl. Acad. Sci. USA.

[18] Hoy J.A., Hargrove M.S. (2008). The structure and function of plant hemoglobins. Plant Physiol. Biochem..

[19] Gisbert C., Timoneda A., Porcel R., Ros R., Mulet J.M. (2020). Overexpression of BvHb2, a Class 2 Non-Symbiotic Hemoglobin from Sugar Beet, Confers Drought-Induced Withering Resistance and Alters Iron Content in Tomato. Agronomy.

[20] Ivarson E., Leiva-Eriksson N., Ahlman A., Kanagarajan S., Bülow L., Zhu L.-H. (2017). Effects of Overexpression of WRI1 and Hemoglobin Genes on the Seed Oil Content of Lepidium campestre. Front. Plant Sci..

[21] Holmberg N., Lilius G., Bailey J.E., Bülow L. (1997). Transgenic tobacco expressing Vitreoscilla hemoglobin exhibits enhanced growth and altered metabolite production. Nature Biotechnol..

[22] Bülow L. (1999). The metabolic effects of native and transgenic hemoglobins on plants. Trends Biotechnol..

[23] Christensen S., Groth L., Leiva-Eriksson N., Nyblom M., Bülow L. (2022). Oxidative Implications of Substituting a Conserved Cysteine Residue in Sugar Beet Phytoglobin BvPgb 1.2. Antioxidants.

[24] Marion D. (2013). An introduction to biological NMR spectroscopy. Mol. Cell. Proteom..

[25] Bax A. (1994). Multidimensional nuclear magnetic resonance methods for protein studies. Curr. Opin. Struct. Biol..

[26] Bax A., Grzesiek S. (1993). Methodological advances in protein NMR. Acc. Chem. Res..

[27] Kay L.E., Gardner K. (1997). Solution NMR spectroscopy beyond 25 kDa. Curr. Opin. Struct. Biol..

[28] Clore G.M., Gronenborn A. (1998). Determining the structures of large proteins and protein complexes by NMR. Trends Biotechnol..

[29] Wüthrich K., Hochmann J., Keller R., Wagner G., Brunori M., Giacometti C. (1975). 1H NMR relaxation in high-spin ferrous hemoproteins. J. Magn. Reson..

[30] Banci L., Bertini I., Marconi S., Pierattelli R. (1993). 1H-NMR study of reduced heme proteins myoglobin and cytochrome P450. Eur. J. Biochem..

[31] Zhao J., Xue M., Gudanis D., Gracz H., Findenegg G.H., Gdaniec Z., Franzen S. (2018). Dynamics of dehaloperoxidase-hemoglobin A derived from NMR relaxation spectroscopy and molecular dynamics simulation. J. Inorg. Biochem..

[32] Laine J.M., Amat M., Morgan B.R., Royer J.W.E., Massi F. (2014). Insight into the Allosteric Mechanism of Scapharca Dimeric Hemoglobin. Biochemistry.

[33] Hargrove M.S., Brucker E.A., Stec B., Sarath G., Arredondo-Peter R., Klucas R.V., Olson J.S., Phillips G.N. (2000). Crystal structure of a nonsymbiotic plant hemoglobin. Structure.

[34] Sturms R., Kakar S., Trent J., Hargrove M.S. (2010). Trema and Parasponia Hemoglobins Reveal Convergent Evolution of Oxygen Transport in Plants. Biochemistry.

[35] Kakar S., Sturms R., Tiffany A., Nix J.C., DiSpirito A.A., Hargrove M.S. (2011). Crystal structures of Parasponia and Trema hemoglobins: Differential heme coordination is linked to quaternary structure. Biochemistry.

[36] Astegno A., Conter C., Bertoldi M., Dominici P. (2020). Structural Insights into the Heme Pocket and Oligomeric State of Non-Symbiotic Hemoglobins from Arabidopsis thaliana. Biomolecules.

[37] Savard P.-Y., Daigle R., Morin S., Sebilo A., Meindre F., Lagüe P., Guertin M., Gagné S.M. (2011). Structure and Dynamics of Mycobacterium tuberculosis Truncated Hemoglobin N: Insights from NMR Spectroscopy and Molecular Dynamics Simulations. Biochemistry.

[38] Song X.J., Yuan Y., Simplaceanu V., Sahu S.C., Ho N.T., Ho C. (2007). A Comparative NMR Study of the Polypeptide Backbone Dynamics of Hemoglobin in the Deoxy and Carbonmonoxy Forms. Biochemistry.

[39] Volkman B.F., Alam S.L., Satterlee J.D., Markley J.L. (1998). Solution Structure and Backbone Dynamics of Component IV Glycera dibranchiata Monomeric Hemoglobin−CO. Biochemistry.

[40] Fushman D., Varadan R., Assfalg M., Walker O. (2004). Determining domain orientation in macromolecules by using spin-relaxation and residual dipolar coupling measurements. Prog. Nucl. Magn. Reson. Spectrosc..

[41] Wang D., Kreutzer U., Chung Y., Jue T. (1997). Myoglobin and hemoglobin rotational diffusion in the cell. Biophys. J..

[42] Mukhi N., Dhindwal S., Uppal S., Kumar P., Kaur J., Kundu S. (2013). X-Ray crystallographic structural characteristics of Arabidopsis hemoglobin I and their functional implications. Biochim Biophys Acta Proteins and Proteomics..

[43] Silkstone R.S., Silkstone G., Baath J.A., Rajagopal B., Nicholls P., Reeder B.J., Ronda L., Bulow L., Cooper C.E. (2016). The βLys66Tyr Variant of Human Hemoglobin as a Component of a Blood Substitute. Oxygen Transport to Tissue XXXVII.

[44] Reeder B.J., Grey M., Silaghi-Dumitrescu R.-L., Svistunenko D.A., Bülow L., Cooper C.E., Wilson M.T. (2008). Tyrosine residues as redox cofactors in human hemoglobin: Implications for engineering nontoxic blood substitutes. J. Biol. Chem..

[45] Opitz C., Ahrné E., Goldie K.N., Schmidt A., Grzesiek S. (2018). Deuterium induces a distinctive Escherichia coli proteome that correlates with the reduction in growth rate. J. Biol. Chem..

[46] Jaravine V.A., Orekhov V.Y. (2006). Targeted acquisition for real-time NMR spectroscopy. J. Am. Chem. Soc..

[47] Kazimierczuk K., Orekhov V.Y. (2011). Accelerated NMR spectroscopy by using compressed sensing. Angew. Chem. Int. Ed..

[48] Mayzel M., Rosenlöw J., Isaksson L., Orekhov V.Y. (2014). Time-resolved multidimensional NMR with non-uniform sampling. J. Biomol. NMR.

[49] Vranken W., Boucher W., Stevens T.J., Fogh R.H., Pajon A., Llinas M., Ulrich E.L., Markley J.L., Ionides J., Laue E.D. (2005). The CCPN data model for NMR spectroscopy: Development of a software pipeline. Proteins: Struct. Funct. Bioinform..

[50] Zhu G., Xia Y., Nicholson L.K., Sze K.H. (2000). Protein dynamics measurements by TROSY-based NMR experiments. J. Magn. Reson..

[51] Delaglio F., Grzesiek S., Vuister G.W., Zhu G., Pfeifer J., Bax A. (1995). NMRPipe: A multidimensional spectral processing system based on UNIX pipes. J. Biol. NMR..

[52] Ahlner A., Carlsson M., Jonsson B.-H., Lundström P. (2013). PINT: A software for integration of peak volumes and extraction of relaxation rates. J. Biol. NMR..

[53] De la Torre J.G., Huertas M., Carrasco B. (2000). HYDRONMR: Prediction of NMR relaxation of globular proteins from atomic-level structures and hydrodynamic calculations. J. Magn. Reson..

[54] Maciejewski M.W., Schuyler A.D., Gryk M.R., Moraru I.I., Romero P.R., Ulrich E.L., Eghbalnia H.R., Livny M., Delaglio F., Hoch J.C. (2017). NMRbox: A Resource for Biomolecular NMR Computation. Biophys. J..

[55] Walker O., Varadan R., Fushman D. (2004). Fushman, Efficient and accurate determination of the overall rotational diffusion tensor of a molecule from 15N relaxation data using computer program ROTDIF. J. Magn. Reson..

[56] Berlin K., Longhini A., Dayie T.K., Fushman D. (2013). Deriving quantitative dynamics information for proteins and RNAs using ROTDIF with a graphical user interface. J. Biol. NMR..

[57] Pettersen E.F., Goddard T.D., Huang C.C., Couch G.S., Greenblatt D.M., Meng E.C., Ferrin T.E. (2004). UCSF Chimera—A visualization system for exploratory research and analysis. J. Comp. Chem..

